# The Sobering Sting: Oleoyl Serotonin Is a Novel *Stephanoconus* Snail Venom-Derived Antagonist of Cannabinoid Receptors That Counteracts Learning and Memory Deficits

**DOI:** 10.3390/biomedicines12020454

**Published:** 2024-02-18

**Authors:** Dongchen An, Guilherme Salgado Carrazoni, Ben-Hur Souto das Neves, Rudi D’Hooge, Steve Peigneur, Jan Tytgat

**Affiliations:** 1 Toxicology and Pharmacology, KU Leuven, Campus Gasthuisberg, ON2, Herestraat 49, Box-922, 3000 Leuven, Belgium; 2 Laboratory of Biological Psychology, KU Leuven, Tiensestraat 102, Box-3714, 3000 Leuven, Belgium; guilhermecarrazoni@gmail.com (G.S.C.); benhur.neves@hotmail.com (B.-H.S.d.N.); rudi.dhooge@kuleuven.be (R.D.)

**Keywords:** oleoyl serotonin, cannabinoid receptor, blocker, learning and memory

## Abstract

Cannabinoid receptors (CB1 and CB2) are promising targets for a better understanding of neurological diseases. Nevertheless, only a few ligands of CB have reached clinical application so far. Venoms are considered as interesting sources of novel biologically active compounds. Here, we describe an endocannabinoid-like molecule, oleoyl serotonin (OS), present in the venom of Stephanoconus snails. Using electrophysiological assays, it was shown that OS inhibits CB1 and CB2. Structure–activity relationship studies using a chimeric CB1/2 revealed that the domain encompassing the transmembrane helix V (TMHV)– intracellular loop 3 (ICL3)–TMHVI of the CB2 is critical for the binding and function of OS. We concluded that OS binds to sites of the CB2 that are different from the binding sites of the non-selective CB agonist WIN55,212-2. Behavioral assays in mice showed that OS counteracted learning and memory deficits caused by WIN55,212-2. Furthermore, a selectivity screening of OS showed high selectivity for CB over various ion channels and receptors. Overall, OS may represent a new approach to the prevention and treatment of learning and memory cognition impairment in neurological diseases.

## 1. Introduction

Cannabinoid (CB) receptors (CB1 and CB2) are members of the endocannabinoid system (ECS) that also comprises endocannabinoids such as anandamide (AEA) and 2-arachidonoylglycerol (2-AG) and specific enzymes involved in endocannabinoid synthesis and degradation [1]. Endocannabinoid signaling is involved in the regulation of cell, tissue, organ, and organism homeostasis, brain development, neurotransmitter release and synaptic plasticity, and cytokine release from microglia [1]. CB receptors, one of the key players and the target of endocannabinoids in the ECS, belong to the family of seven transmembrane G protein-coupled receptors (GPCRs). 

There are two main types of CB receptors; the CB1 receptor is claimed to be the most abundant GPCR in the mammalian brain, with the highest concentrations demonstrated in the basal ganglia, cerebellum, hippocampus, and cerebral cortex [1,2]. It also contributes to the regulation of movement, coordination, cognition (learning and memory), nociception, appetite, sensory perception, and processing of reward and emotions [2,3]. CB2 receptor expression was initially reported to be limited to peripheral immune cells, such as macrophages [4]. However, subsequent research revealed that CB2 receptors are also present in neurons, of which the mRNA is 100–200 times less abundant than CB1 receptor mRNA [5]. This finding has led to numerous discoveries into the role of CB2 receptors in neural functions, including nociception, neurogenesis, and neuroimmune modulation [3,4,6]. 

The physiological CB receptor signaling involves many aspects. As GPCRs, CB receptor activation evoked by cannabinoids typically engages G protein coupling [4,6]. The α subunit of the G protein (mainly Gi/o) (Gα) replaces its bound GDP with GTP [4,6], which leads to the separation of the βγ subunit (Gβγ) from the α subunit. The separated G protein subunits then act as downstream effectors to enhance the receptor-mediated signal [4,6]. Specifically, Gβγ directly binds to and activates G protein-coupled inwardly rectifying potassium (GIRK) channels [4,6]. For example, the binding of agonists such as the endogenous AEA and the synthetic WIN55,212-2 to CB receptors predominantly stimulates Gi/o pathways, resulting in downstream activation of GIRK channels [7,8,9]. 

The structural analysis of activation mechanisms of CB receptors reveals that the movements of the transmembrane alpha (TMH) V and TMHVI are important in the activation processes of cannabinoid receptors. An outward movement of TMHVI is suggested as a characteristic of cannabinoid receptor activation, and an extension of TMHV can result in new interactions with Gi(α) [10]. These structural changes allow the G protein to engage with the receptor core [10]. 

Though over a hundred phytocannabinoids have been found and numerous synthetic cannabinoids have been generated over the past few decades, only a few phytocannabinoids and synthetic phytocannabinoid analogs have reached the clinical market so far [3,11,12]. For example, the approvals of nabiximols, a mixture of tetrahydrocannabinol and the non-psychotropic cannabinoid cannabidiol, for the treatment of spasticity and neuropathic pain in multiple sclerosis, and of purified botanical cannabidiol for the treatment of otherwise untreatable forms of pediatric epilepsy, have brought the therapeutic use of cannabinoids and endocannabinoids in neurological diseases into the limelight [3]. As all the therapeutics targeting CB receptors are synthetically derived from phytocannabinoids, natural products represent the major source for finding potential drugs targeting CB receptors. 

Among natural products, animal venoms have risen as a promising source of lead compounds for ion channel and receptor drug discovery [13]. They comprise numerous toxin families, mainly peptides and proteins, and small molecules [14]. During evolution, toxins acquired the ability to bind selectively and with high affinity to different biological targets, e.g., GPCRs and ion channels [13], which enables them to affect various vitally essential systems of the prey and result in immobilization or death [14]. 

Cone snails belong to the family Conidae and they are venomous sea snails. All cone snails have a venom gland that produces complex venoms, each with a distinctive mixture of, typically, 100–200 bioactive venom components [15]. Cone snails use their venoms to capture prey, defend against predators, and for competitive interactions with other snails [15]. Prior characterization of cone snail venoms established that bioactive venom components are relatively small, structured peptides (10–35 amino acids), most with multiple disulfide crosslinks [16]. These peptides have been widely studied in many laboratories, leading to pharmaceutical agents and probes, such as Prialt^®^ (also called Ziconotide), which is an intrathecal analgesic medication used for the treatment of chronic pain and is the synthetic form of an ω-conotoxin peptide found in the venom of Conus magus [17,18,19,20]. 

Much less studied is the emerging evidence that specific cone snail lineages use non-peptidic small molecules as part of their envenomation strategy [21]. These small molecules also play a role in capturing prey and expanding the molecular diversity of cone snail venom beyond peptides [21,22,23,24]. After discovering genuanine, a derivative of guanine and the first bioactive venom-derived small molecule [23], researchers have investigated numerous cone snail venoms for non-peptidic bioactive components. The venoms of a group of cone snails called Stephanoconus, which feed on polychaetes, contain not only genuanine but also many other small molecules [21]. These non-peptidic compounds have been shown to act on neurons and may have the potential to treat neuronal diseases. Conazolium A, discovered in the deep-water snail C. imperialis, is a competitive antagonist of the human α7 nicotinic acetylcholine receptor (hα7-nAChR) in neurons [21,24,25,26]. Like venom peptides, these small molecules may offer new possibilities for developing pharmacological agents. 

**Figure 1:**
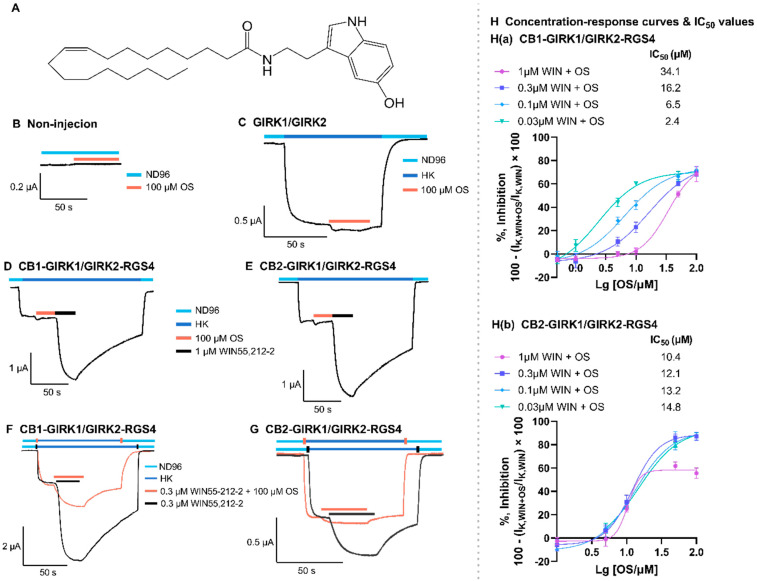
The effect of OS on non-injected oocytes, GIRK1/GIRK2 channels, and CB receptors. (**A**) The structure of OS. (**B**–**F**) Representative electrophysiological current traces showing the effect of OS on ion currents in non-injected oocytes and oocytes expressing receptors and/or ion channels. (**B**) In total, 100 μM OS in the presence of ND96 was applied to non-injected oocytes and did not change the current response through the oocytes. (**C**) In total, 100 μM OS in the presence of HK was applied to GIRK1/GIRK2 channels expressed in oocytes following I_K,basal_ and did not remarkably change the response of I_K,basal_. (**D**,**E**) In total, 100 μM OS was applied to CB1-GIRK1/GIRK2-RGS4 and CB2-GIRK1/GIRK2-RGS4 coupling systems expressed in oocytes for 20–30 s following I_K,basal_ and did not change the response of I_K,basal._ This OS application was followed by 1 μM WIN55,212-2 that induces CB-dependent inward K^+^ currents upon I_K,basal_ (I_K,WIN_). (**F**,**G**) In total, 0.3 μM WIN55,212-2 + 100 μM OS (orange trace) and 0.3 μM WIN55,212-2 alone (black trace) were applied to CB1-GIRK1/GIRK2-RGS4 and CB2-GIRK1/GIRK2-RGS4 coupling systems expressed in oocytes for 20–30 s following I_K,basal_. The response of I_K,WIN+OS_ is notably smaller than I_K,WIN_. All panels are representative of at least three independent experiments (*n* ≥ 3). (**H**(**a**),**H**(**b**)) The OS concentration–current response curves of blocking CB1 and CB2 receptors, respectively, activated by different concentrations of WIN55,212-2. All experiment data points are representative of at least three independent experiments (*n* ≥ 3) and data are plotted as mean ± SD.

Oleoyl serotonin (OS) is a small molecule and a neurotransmitter abundantly found in the venom of shallow-water cone snails—Stephanoconus snails [24]. The records in the literature suggest that the Stephanoconus snails have a cosmopolitan distribution and have specialized on amphinomid polychaetes as their major prey (a group widely known as fireworms, which have painful stinging bristles) [21]. Regarding the structure, OS (Figure 1A) shares a similar structure with AEA (a partial agonist of CB1 receptors) and Org27569 (a positive allosteric modulator of CB1 receptors in binding, a negative allosteric modulator of CB1 receptors in function) (Figure S1), which indicates that it may act through CB receptors and, as seen in other studies that modulated CB receptors’ function, affect behavior and memory [1,2,3].

Venoms can be seen as an untapped source of molecules that can be highly selective and potent ligands for a wide range of ion channels and receptors [27,28]. In the present study, we evaluated the effects of an endocannabinoid-like molecule found in cone snail venom. We found that OS is a selective blocker of CB receptors. First, we utilized the two-electrode voltage-clamp technique to investigate the activity of OS on the system of CB receptors coupled to G protein-coupled inwardly rectifying potassium (GIRK1/GIRK2) channels and regulator G protein signaling 4 (RGS4) expressed in Xenopus oocytes. A selectivity screening to examine potential off-target effects revealed a high selectivity of OS for CB receptors. Furthermore, structure–activity relationship studies provided insights into the molecular mechanisms of the interaction between OS and CB receptors. Finally, our in vivo evaluation of behavioral assays in mice suggests that OS counteracted the learning and memory deficit caused by WIN55,212-2 without severe side effects on locomotion and exploratory and anxiety-like behavior.

## 2. Materials and Methods

Animal experiments were approved by the Animal Ethics Committee of KU Leuven (Project No. 020/2023 and P186/2019) in accordance with EU Council Directive 2010/63/EU. Detailed descriptions of animals and reagents and methods for in vitro transcription of cDNA, plasmid construction, isolation of oocyte, heterologous expression, electrophysiological measurements, behavioral experiments, and statistical analysis are as follows:

In vitro transcription of cDNA clones. The plasmids were linearized with XhoI (for hCB1-pGEMHE, hCB2-pGEMHE, human histamine receptor type 4 (hH4R)-pcDNA3.1+, and human mas-related G protein-coupled receptor member X2 (hMRGPRX2)-pcDNA3.1+), EcoRI (for mGIRK1-pSPOR), SaII (for mGIRK2-pBScMXT), NheI (for hRGS4-pGEMHE and the mouse serotonin receptor type 4a (m5-HT4aR)-pSGEM), NheI (for the human μ-opioid receptor (hMOR)-pGEMHE), BamHI (for hα7-nAChR-pMTX), XbaI (for the Transient receptor potential vanilloid 1 (hTRPV1)-pGEMHE), SpeI (for human voltage-gated potassium channel 7.1 (K_V_7.1)-pGEMHE). For the expression of receptors and channels in Xenopus laevis oocytes, the linearized plasmids of the channel/subunit were transcribed using the T7 (for hCB1, hCB2, m5-HT4aR, hH4R, hMOR, hMRGPRX2, hTRPV1, and hK_V_7.1), SP6 (for mGIRK1 and hα7-nAChR), and T3 (for mGIRK2) mMESSAGE mMACHINE transcription kits (Ambion, Austin, TX, USA). The synthesis of mIRK1 cRNA was previously performed in our lab.

Plasmid construction of chimeric CB1/2 receptors. The chimeric CB1/2 receptor was designed to replace the transmembrane alpha helix V (TMHV)–intracellular loop 3 (ICL3)–TMHVI fragment of the CB2 receptor with that of the CB1 receptor. This chimera was synthesized and inserted into the XhoI and EcoRI sites of the pGEMHE vector by GenScript (Rijswijk, The Netherlands). The construct of the chimeric CB1/2-pGEMHE plasmid was sequenced to verify that it had the correct sequences and orientations.

Isolation of *Xenopus laevis* oocytes. All procedures for the use and handling of adult female Xenopus laevis frogs (CRB Xénopes, Rennes, France) were approved by the Animal Ethics Committee of the KU Leuven (Project No. P186/2019) following regulations of the European Union (EU) concerning the welfare of laboratory animals as declared in Directive 2010/63/EU. Oocytes were isolated as described previously [29]. Stage V–VI Xenopus laevis oocytes were isolated by partial ovariectomy. The animals were anesthetized by a 15 min submersion in 0.1% tricaine methane sulfonate (Sigma-Aldrich Chemical, St. Louis, MO, USA) solution (pH 7.0). The oocytes were enzymatically defolliculated by collagenase (3 mg/mL) (Sigma-Aldrich Chemical, St. Louis, MO, USA) digestion at 16 °C on a rocker platform in a Ca^2+^-free ND96 solution. Isolated stage V–VI oocytes were then maintained in ND96 solution containing Theophylline and Gentamicin at 16 °C. The ND96 solution comprised 96 mM NaCl, 2 mM MgCl_2_, 2 mM KCl, 5 mM HEPES, and 1.8 mM CaCl2, with a pH of 7.5.

Heterologous expression in *Xenopus laevis* oocytes. On the first day after enzymatic isolation of oocytes (day 1), a mixture of cRNAs dissolved in nuclease-free water at a final injection volume of 50 nL was injected into oocytes (Nanoliter Injector A203XVZ, World Precision Instruments, Sarasota, FL, USA). Oocytes were injected with cRNA mixtures of GPCR (50–75 ng) (hCB1, hCB2, chimeric hCB1/2, m5-HT4aR, hH4R, hMOR, or hMRGPRX2) + mGIRK1 (50–75 ng) + mGIRK2 (50–75 ng) + hRGS4 (~50 ng), or mGIRK1 (50–75 ng) + mGIRK2 (50–75 ng), or the cRNA alone of hα7-nAChR (~50 ng), hTRPV1 (~50 ng), or hK_V_7.1 (~50 ng). 

Following the cRNA injection into oocytes and 2–10 days of incubation at 16 °C (2–3 days for the expression of GIRK1/GIRK2 channels, TRPV1, and α7-nAChR, 4–5 days for the expression of GPCR-GIRK1/GIRK2-RGS4 coupling systems, and 7–10 days for K_V_7.1 channels), electrophysiological experiments were conducted.

Electrophysiological measurements. Ion currents through receptor- and channel-expressed oocytes were measured using a two-electrode voltage-clamp (TEVC) (GeneClamp 500B, Axon Instruments, San Jose, CA, USA). Membrane currents from voltage-clamped oocytes were digitized using a Digidata 1550 low-noise data acquisition system (Axon Instruments, San Jose, CA, USA) and a Dell PC running pCLAMP 10.1 software (Axon Instruments, San Jose, CA, USA).

Injected oocytes were placed in a 0.2 mL recording chamber continuously perfused with ND96 solution comprising 96 mM NaCl, 2 mM MgCl2, 2 mM KCl, 5 mM HEPES, and 1.8 mM CaCl2, with a final pH of 7.5. 

For oocytes expressing GPCR (CB1, CB2, chimeric CB1/2, 5-HT4aR, H4R, MOR, or MRGPRX2)-GIRK1/GIRK2-RGS4 coupling systems, currents were measured using the protocol of −90 mV membrane potential. After electrode impalement and clamping the potential to −90 mV, the perfusion solution was changed from ND96 to high potassium (HK) solution composed of 96 mM KCl, 2 mM NaCl, 1 mM MgCl2, 1.8 mM CaCl_2_, and 5 mM HEPES, with a final pH of 7.5. The HK-evoked increase in inward K+ currents represents a “basal” K+ current (IK,basal). In the presence of HK, the GPCR agonist (0.3 μM WIN 55,212-2 for CB receptors and chimeric CB1/2 receptors, 1 μM serotonin for 5-HT4aR, 0.02 μM histamine for H4R, 0.2 μM morphine for MOR, and 0.2 μM C48/80 for MRGPRX2) (Sigma-Aldrich Chemical, St. Louis, MO, USA) or the GPCR agonist + OS was applied to oocytes expressing the GPCR-GIRK1/GIRK2-RGS4 coupling system for 20–30 s and then washed out by HK. 

For oocytes expressing α7-nAChR, currents were measured by clamping the cells at a membrane potential of −70 mV. Then, 100 µM Acetylcholine (the α7-nAChR agonist) + OS was rapidly applied for 10 s and then washed out. Acetylcholine alone was quickly applied for 10 s and then washed out before and after the application of acetylcholine + OS.

For oocytes expressing TRPV1, currents were measured by clamping the cells at a membrane potential of −90 mV. Then, 1 µM Capsaicin (the TRPV1 agonist) was applied for 20–30 s, followed by the application of capsaicin + OS, and then washed out.

For oocytes expressing K_V_7.1 channels, potassium (K^+^) currents were measured using the protocol of 1.3 s depolarizations to +20 mV from a holding potential of −90 mV. OS was directly applied to the oocytes expressing K_V_7.1 channels. All recordings were performed at room temperature (21–23 °C). 

Animal housing. Forty-eight Male C57BL/6J mice were purchased from Janvier Labs (Le Genest-Saint-Isle, France) and were time-specifically housed in a 12 h light-dark cycle (lights on at 7 am), with ad libitum water and food in conditioned rooms (22 °C, humidity ~60%). 

Behavior assays. All mice were subjected to a behavioral test battery that consisted of several general performance tasks of locomotor activity, exploratory and anxiety-like behavior, and learning and memory. Mice were divided into four groups: Vehicle, OS (2 mg/kg), WIN55 (0.5 mg/kg), or OS + WIN. Each group consisted of 7–14 mice that received two i.p injections before each test, with a 10 min interval between each injection. Veh + Veh were injected with PBS. Veh + WIN were injected with PBS and WIN55. Veh + OS were injected with PBS and OS. OS + WIN were injected with OS and WIN55. PBS was used as vehicle as it was used for dilution of drugs. Tests were performed in the order described below after 10 min of the last injection, with a 24 h interval between each test. 

Cage activity assessment: At P60, animals were moved to the cage activity room for one hour before injections for habituation.. The cage activity assessment consists of placing the mice in a transparent box which is placed between three infrared sensors (two on the cage’s side and one in its front) that count how many times the mice cross the infrared beams. The times of beams crossed in each 30 min were counted over a period of 24 h and used as a locomotor activity measurement [30]. In addition, we also compared the total number of beams crossed during the day and night period. For this test, we used Veh (*n* = 10), OS (*n* = 9), WIN (*n* = 11), OS + WIN (*n* = 8).

Open Field (OF): One day after the cage activity test, mice were tested in the OF paradigm. The OF aimed to test the OS, WIN, and OS + WIN effect on animal locomotion and exploratory behavior. Animals were placed in the test room one hour before starting the test. For the OF, animals were placed in a plexiglass box (60 × 60 × 60 cm) divided into three zones (center, periphery, and corners) for 10 min and left free to explore. Total distance and time in zones were counted [30]. Here, more time in the center meant an anxiolytic effect, and a higher distance walking meant an increase in locomotor activity. For this test, we used Veh (*n* = 8), OS (*n* = 10), WIN (*n* = 7), OS + WIN (*n* = 8).

Elevated Plus Maze (EPM): EPM was used to assess the effects of OS, WIN, and OS + WIN on anxiety-like behavior. One day after the OF test, animals were tested in the EPM. The procedure before the actual test was the same as previous tests. The EPM consisted of a cross with two open and two closed arms 50 cm high from the ground level [31]. To start the test, animals were placed in one of the open arms. Time in open and closed arms was measured over 10 min. Anxiety index (AI) was calculated using the following equation: AI = 1 − [([time the animal remained in the open arms (seconds)/test duration] + [input frequency in the open arms/total number of entries])/2]. Here, more time in the open arms compared to control meant an anxiolytic effect. For this test, we used Veh (*n* = 7), OS (*n* = 10), WIN (*n* = 7), OS + WIN (*n* = 8).

Morris Water Maze (MWM): MWM [32] was used to assess the effects of OS, WIN, and OS + WIN in memory one day after the EPM test. MWM is a gold standard test to assess learning and memory consisting of 10 days of training and two probe trials in a pool with water at a temperature of 26 °C, with one submersed platform invisible to mice. Each mouse could try to find the platform for 100 s, four times per day, with an inter-trial interval of 15–30 min. In each trial, animals were released randomly from each of the four starting positions. This protocol of training was repeated for ten days (training sessions). The platform was removed on the 6th and 11th days, and the animals were left free to swim for 100 s (probe trials). Right after the first probe trial (day 6), animals were trained again with the platform at the same place. During the training sessions, the mean latency to find the platform for the session was calculated for each animal as the average of the four trials of the day. During the probe trials, the pool was divided into four equal virtual quadrants, and the time spent in the target quadrant (TQ) (i.e., the quadrant where the platform was located during the training sessions) was assessed as a measure of memory retention. For this test, we used Veh (*n* = 9), OS (*n* = 8), WIN (*n* = 8), OS + WIN (*n* = 14).

Cage activity was analyzed using property software. OF and EPM were analyzed using AnyMaze v4.7 (Any-Maze, Inc., Dublin, Ireland). MWM was analyzed using EthoVision v15.0 (Noldus, Inc., Wageningen, the Netherlands).

Statistical analysis. Electrophysiological and behavioral data were analyzed using GraphPad Prism 9 software for Windows (GraphPad Software, Inc., San Diego, CA, USA). A two-way ANOVA multiple comparison test with Tukey’s post hoc was used to compare the significance between multiple groups. For all analyses, statistical significance was set at * *p* < 0.05, ** *p* < 0.01, *** *p* < 0.001, **** *p* < 0.0001. All data are shown as mean ± standard deviation (SD).

## 3. Results

### 3.1. Oleoyl Serotonin Blocks CB Receptors Activated by WIN55,212-2 

The application of 1 μM WIN55,212-2 (a non-selective agonist of CB receptors) activated CB1 and CB2 receptors in CB-GIRK1/GIRK2-RGS4 coupling systems. Also, WIN55,212-2, in the presence of HK, induced receptor-dependent inward K+ currents upon I_K,basal (IK,WIN)_ (Figure 1D,E). The EC50 value for WIN55,212.2 to activate CB receptors expressed in oocytes was determined previously in our system (Figure S2) [33]. Additionally, 100 μM OS inhibited 0.3 μM WIN55,212-2-induced inward K^+^ currents (I_K,WIN+OS_) in CB-GIRK1/GIRK2-RGS4 coupling systems when they were co-applied (Figure 1F,G). To rule out non-specific interactions, OS was also tested on non-injected oocytes and oocytes expressing GIRK1/GIRK2 channels. Here, 100 μM OS did not show activity on non-injected oocytes (Figure 1B). Similarly, applying 100 μM OS to oocytes expressing only GIRK1/GIRK2 channels did not significantly change I_K,basal_ (Figure 1C).

### 3.2. OS Exhibits Selectivity for CB Receptors over Other Molecular Targets

The OS compound was initially examined for its ability to modulate CB receptors. To assess specificity, OS was screened against ion channels and receptors. First, its selectivity for other GPCRs, namely 5-HT4aR, H4R, MOR, and MRGPRX2, was investigated. The inclusion of α7-nAChR in this selectivity screening was also crucial, based on its significant role as a modulator in essential brain functions such as memory, learning, and attention, similar to CB receptors [34]. Furthermore, it is reported that OS displayed antagonist properties against TRPV1 channels overexpressed in HEK cells [35]. K_V_7.1 channels are a target of the endocannabinoid AEA [36], which possesses structural similarities like the OS compound, as illustrated in Figure S1. Na_V_1.5, K_V_1.5, and K_V_11.1 (hERG) were tested because they are critical for cardiac safety.

At a concentration of 100 μM, OS did not show significant activity on 5-HT4aR, H4R, MOR, MRGPRX2, α7-nAChR, TRPV1, Ca_V_3.1, Na_V_1.5, hERG, K_V_1.5, and K_V_7.1 expressed in oocytes (Figure S3). 

OS was shown to function as an antagonist of TRPV1 with an IC50 value of 2.57 μM using HEK cells overexpressing TRPV1 [35]. However, in our study, OS did not show significant activity on TRPV1, probably due to the different measuring systems. The binding sites on the TRPV1 channels for OS remain unrevealed, and further studies are required to fully understand the mechanisms. 

### 3.3. Pharmacological Mechanisms of Oleoyl Serotonin-Induced Blockade of CB Receptors 

To explore the pharmacological mechanisms of OS-induced blockade of CB receptors activated by WIN55,212-2, a range of different concentrations of OS were co-applied with diverse concentrations of WIN55,212-2 to CB-GIRK1/GIRK2-RGS4 coupling systems expressed in oocytes. A more potent inhibition on WIN55,212-2-induced currents by OS is observed in the presence of lower concentrations of WIN55,212-2. Such a growing IC50 value is a characteristic of competitive antagonism (Figure 1H(a)). On the other hand, the inhibition on WIN55,212-2-induced currents by OS is similar at all concentrations of WIN55,212-2, showing a stable IC50 value that is characteristic of non-competitive antagonism (Figure 1H(b)). The lowest IC50 values of OS inhibiting the CB1 and the CB2 receptors are 2.4 μM (95% confidence interval (CI): 1.6–3.5 μM) and 10.4 μM (95%CI: 9.1–12.5 μM), respectively.

### 3.4. The TMHV-ICL3-TMHVI Domain of CB Receptors Is the Binding Region for Oleoyl Serotonin

**Figure 2:**
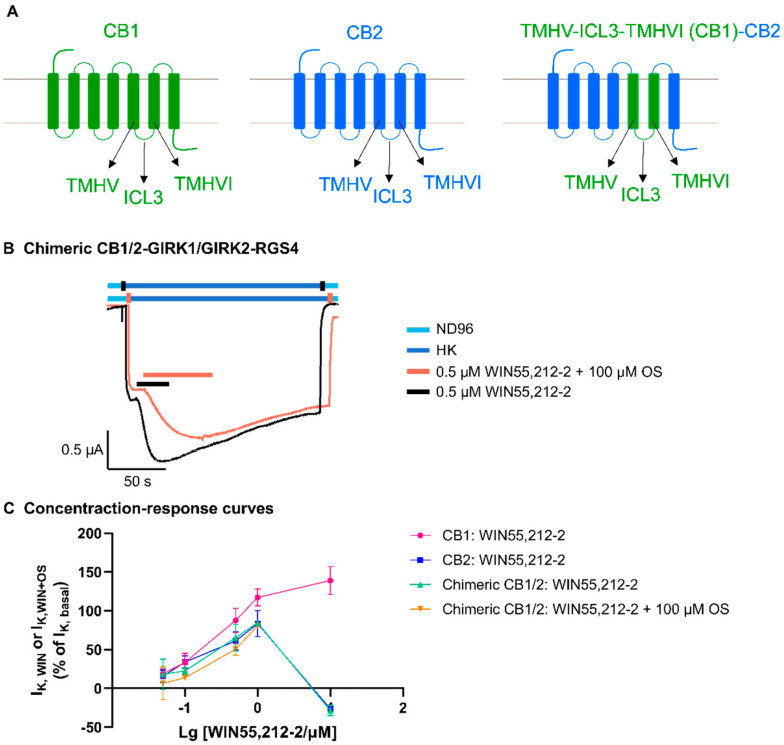
The effect of OS compound on the chimeric CB1/2 receptor. (**A**) Sketches of the CB1, CB2, and the chimeric CB1/2 structures. (**B**) Representative electrophysiological current traces were recorded for the application of 0.5 μM WIN55,212-2 + 100 μM OS (orange trace) and 0.5 μM WIN55,212-2 (black trace) to chimeric CB1/2-GIRK1/GIRK2-RGS4 coupling systems expressed in oocytes following I_K,basal_. OS did not change the response of WIN55,212-2-induced receptor-dependent inward K^+^ currents upon I_K,basal_ (I_K,WIN_). This panel is representative of at least three independent experiments (*n* ≥ 3). (**C**) WIN55,212-2 concentration–current response curves for activating and blocking CB1, CB2, and chimeric CB1/2 receptors in coupling systems expressed in oocytes. All experiment data points are representative of at least three independent experiments (*n* ≥ 3) and data are plotted as mean ± SD.

To identify if the TMHV-ICL3-TMHVI domain is responsible for the OS-induced blockade of CB receptors, a chimeric CB1/2 receptor was constructed in which the TMHV-ICL3-TMHVI domain of the CB2 receptor was replaced with the homologous segment of the CB1 receptor (Figure 2A). These chimeric receptors were then co-expressed with GIRK1/GIRK2 channels and RGS4 proteins in oocytes and investigated for expression and ability to modulate GIRK1/GIRK2 channels. The chimeric CB1/2 receptor coupled to the G protein pathway and stimulated GIRK1/GIRK2 channels via the binding of WIN55,212-2. Interestingly, in the chimeric CB1/2-GIRK1/GIRK2-RGS4 coupling system, the response of I_K,WIN_ is at a similar level as the response of I_K,WIN_ in the CB2-GIRK1/GIRK2-RGS4 coupling system as shown in Figure 2B. This indicates that this chimera was utterly defective in binding OS to block WIN55,212-2-induced inward K^+^ currents through GIRK1/GIRK2 channels upon I_K,basal_. These results demonstrate that the TMHV-ICL3-TMHVI domain of the CB2 receptor is responsible for the recognition of OS and the OS-induced blockade of CB2 receptors. 

Furthermore, the dual modulation of chimeric CB1/2 and GIRK1/GIRK2 channels by WIN55,212-2 is shown in Figure 2C. The WIN55,212-2 concentration–current response curves for activating and blocking CB2 (in blue) and chimeric CB1/2 receptors (in green) are highly overlapping (Figure 2C), indicating that the dual modulatory effect on the chimera and GIRK1/GIRK2 channels by WIN55,212-2 is highly similar to that observed on CB2 receptors and GIRK1/GIRK2 channels, as described before [33]. 

### 3.5. In Vivo Behavior, Learning, and Memory Effects of Oleoyl Serotonin

An extensive test battery enabled us to document the broad profile of behavioral changes in mice subjected to OS, WIN55,212-2 (WIN), and OS + WIN compounds exposure. We probed the efficacy of the compounds in tests of home cage activity, exploratory and anxiety-like behavior, learning, and memory. 

**Figure 3:**
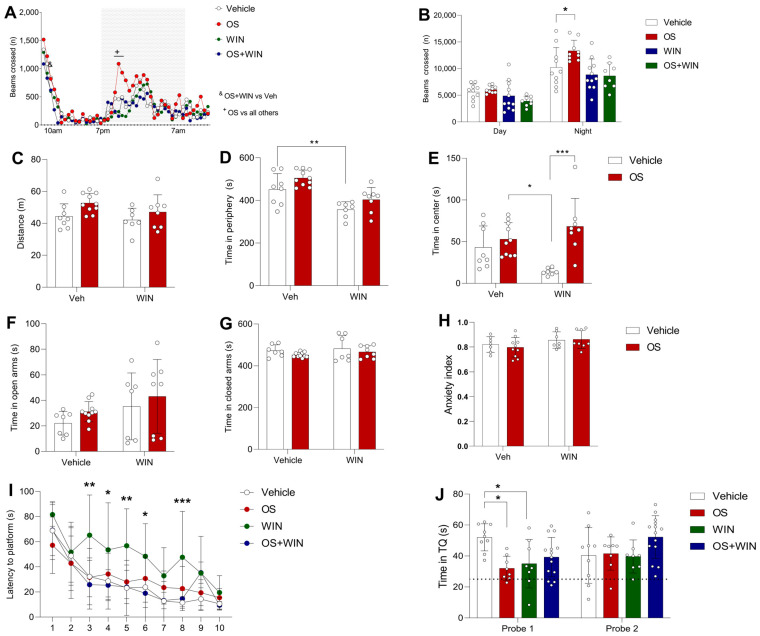
Behavior impact of OS (2 mg/kg), WIN (0.5 mg/kg), or OS + WIN i.p injections 10 min before each test. (**A**,**B**): Cage activity of C57BL/6J adult mice. (**A**) The activity profile revealed changes in beam crossings by mice during the 24 h (Day phase: 7 a.m.–7 p.m., Night phase: 7 p.m.–7 a.m.). (**B**) Assessment of the total amount of beam crossings by mice during the 24 h home cage activity test, divided into day and night phases (*n* = 8–11 mice per group). Data are presented as means ± SD. &: OS + WIN vs. Vehicle, * *p* < 0.05. +: OS vs. all other groups, ** *p* < 0.01. (**C**–**E**): OF test activity of C57BL/6J adult mice. (**C**) Total distance walked by mice in an open field. (**D**) Total time mice spent in the center of an open field. (**E**) Total time mice spent in the periphery of an open field (*n* = 8–11 mice per group). Data are presented as means ± SD. ** *p* < 0.01, the difference between the time of WIN group spent and the control spent in the periphery. (**F**,**G**): EPM test activity of C57BL/6J adult mice after i.p injections. Total time adult C57BL/6J mice spent in open (**F**,**G**) and closed arms in the EPM. There was no significant difference observed between the groups (*n* = 7–11 mice per group). Data are presented as means ± SD. (**H**) Anxiety index of mice during the EPM test. (**I**,**J**): Spatial learning and memory retention activity of C57BL/6J adult mice in the MWM test. (**I**) Total time mice spent to find the platform across ten training days in the MWM. Significant difference was observed on days 3, 4, 5, 6, and 8 between WIN group and the control, * *p* < 0.05, ** *p* < 0.01, and *** *p* < 0.001. (**J**) Total time mice spent in the TQ during probe trials. There is a significant difference between the control and the OS and WIN groups in the probe 1 trial (*n* = 8–15 mice per group). Data are presented as means ± SD, * *p* < 0.05 compared with the vehicle group.

We began with a home cage motor activity test involving wild-type C57BL/6J mice. A two-way RM ANOVA showed the effect for time (F_(13,34, 453,6)_ = 36.36; *p* < 0.0001), treatment (F_(3, 34)_ = 4.798; *p* < 0.0001), and interaction of factors (F(_138, 1564)_ = 2.216; *p* < 0.0001; Figure 3A). We found that 2 h after the beginning of night phase (i.e., 21 h), OS notably increased cage activity compared to all other groups (*p* < 0.05 OS vs. all other groups; Figure 3A). On the other hand, WIN did not change the activity of mice in the same period. Interestingly, OS + WIN injection decreased activity 1 h after injection (*p* = 0.027; Figure 3A) compared to the control group. 

When we calculated the overall locomotion during the night, the two-way RM ANOVA showed an effect for treatment (F_(3, 68)_ = 7.001; *p* = 0.0004). We observed that the OS group moved more in the cage compared to the control group (*p* = 0.031; Figure 3B), showing an increase in home cage activity during the period in which mice already tend to move more inside the cage. The observed effect of the increase in activity for the OS group during the night is mainly due to the increased activity during the first hours of the dark period compared to the control group (*p* = 0.007; Figure 3B). 

When we evaluated the groups on the Open Field (OF) paradigm, we observed no difference between the compound groups and controls on the total distance walked (F_(1, 29)_ = 0.354; *p* = 0.556; Figure 3C), which shows that neither OS nor WIN affected locomotion in our study. However, the two-way RM ANOVA showed an effect for treatment in the time spent in the center zone (F_(1, 29)_ = 14.96; *p* = 0.0006) and in the periphery (F_(1, 29)_ = 7.276; *p* = 0.011). The OS-injected groups spent more time than the WIN-only group in the center zone (OS + Veh vs. Veh + WIN: *p* = 0.011; OS + WIN vs. Veh + WIN: *p* = 0.0006; Figure 3D), and the WIN-injected mice spent less time in the periphery compared to the control (*p* = 0.008; Figure 3E) and OS group (*p* < 0.0001; Figure 3E), but OS injection prevented this decrease (*p* = 0.334; Figure 3E). 

When we evaluated the anxiety-like behavior, we observed that the OS, WIN, and OS + WIN compounds did not exhibit significant effects in wild-type mice, as shown by time spent in the open (F_(1, 28)_ = 0.0092; *P* = 0.924; Figure 3F) and closed arms (F_(1, 28)_ = 0.028; *p* = 0.866; Figure 3G) and the anxiety index (F_(1, 28)_ = 0.3373; *p* = 0.566; Figure 3H). 

Notably, in the Morris water maze (MWM) test, the two-way RM ANOVA showed effects for the time (F_(9, 306)_ = 36.07; *p* < 0.0001; Figure 3I), treatment (F_(3, 34)_ = 6.170; *p* = 0.0018; Figure 3I), and interaction of factors (F_(27, 306)_ = 1.834; *p* = 0.0018; Figure 3I). On day 3 (*p* = 0.002; Figure 3I), day 4 (*p* = 0.041; Figure 3I), day 5 (*p* = 0.002; Figure 3I), day 6 (*p* = 0.041; Figure 3I), and day 8 (*p* = 0.0009; Figure 3I), the WIN-injected mice performed significantly worse than the control, taking more time to find the platform, showing learning and memory deficit (Figure 3H). Pre-injection with OS notably counteracted this deficit over these days, with the OS + WIN group finding the platform on a similar amount of time as the controls (*p* > 0.05 for Veh vs. OS on days 3, 4, 5, 6 and 8; Figure 3I). There was no difference between all groups on days 2 and 10 (*p* > 0.05 on both days; Figure 3I). These results indicate that WIN caused learning and memory deficit until the first probe trial (day 6), and pre-injection of OS can counteract this effect. There was no deficit for WIN in the second probe trial, presumably because WIN had effects until the first probe trial, and then a desensitization of the CB receptors began [37].

In the first probe trial, the two-way RM ANOVA showed interaction of factors (F_(3, 35)_ = 4.650; *p* = 0.007; Figure 3J), and although the OS group performed worse than the control (*p* = 0.01; Figure 3J), the OS-injected mice still spent more than 25 s in the target quadrant (TQ) (*p* = 0.001; Figure 3J). The pool was divided into four quadrants for training (four different start points) and probe (target, opposite, adjacent one, adjacent two); this shows that they remembered where the platform was during the first five days of training, which was not the case for the WIN group (~25%; *p* = 0.11; Figure 3J).

## 4. Discussion

In this study, we found that OS inhibits CB receptors in a concentration-dependent manner, while not showing significant activity on the GIRK1/GIRK2 channel, 5-HT4aR, H4R, MOR, MRGPRX2, α7-nAChR, TRPV1, Ca_V_3.1, Na_V_1.5, hERG, Kv1.5, and K_V_7.1 channel. Interestingly, the antagonism of OS showed different patterns in the two isoforms of CB receptors. OS competitively blocked CB1 receptors, while non-competitively blocked CB2 receptors. We hypothesize that OS binds to the orthosteric binding site of the CB1 receptor that highly overlaps with those of WIN55,212-2. Moreover, OS binds to the binding sites of the CB2 receptor differently from those for the binding of WIN55,212-2. To the best of our knowledge, this is the first report of a small molecule found in animal venom that blocks CB1 and CB2 receptors in competitive and non-competitive ways, respectively. 

To study the structure–activity relationship between OS and the CB receptors, we designed the chimeric CB1/2 receptor, in which the TMHV-ICL3-TMHVI domain of the CB2 receptor was replaced with the corresponding domain of the CB1 receptor. As a result, the antagonism of OS was abolished on the chimeric CB1/2 receptor, which indicates that the TMHV-ICL3-TMHVI domain of the CB2 receptor is critical for the binding and function of OS. 

**Figure 4:**
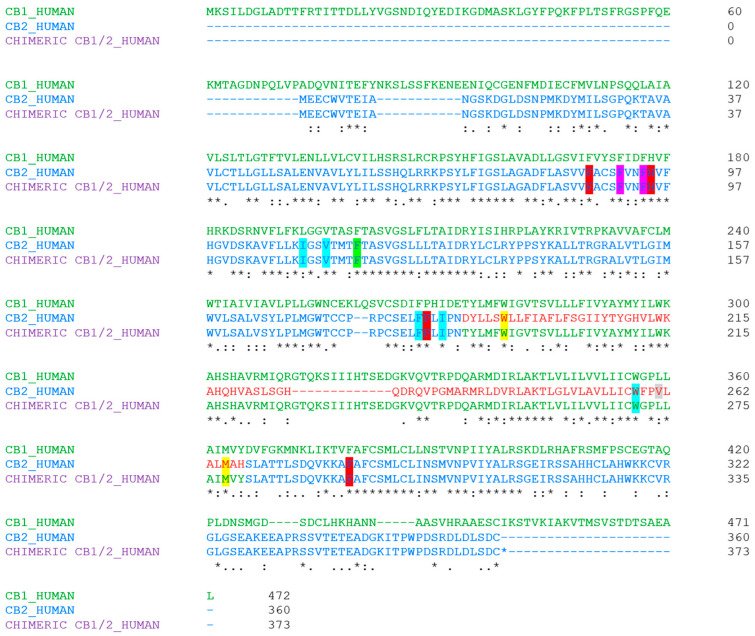
Amino acid sequence alignment of CB1 (in green) and CB2 (in blue) receptors and the chimeric CB1/2 (in blue + green) generated by a standard protein Multiple Sequence Alignment (Clustal Omega). The * indicates identical residues. The TMHV-ICL3-TMHVI domain in the CB2 receptor is in red and the TMHV-ICL3-TMHVI domain in the chimeric CB1/2 is in green. F91 and F94 in both the CB2 and the chimera are highlighted in fuchsia; F87, H95, and P184 in both the CB2 and the chimera, as well as F281 in the CB2 and its corresponding F294 in the chimera are highlighted in red; F117 in both the CB2 and the chimera, as well as W258 in the CB2 and its corresponding W271 in the chimera are highlighted in green; I110, V113, and F183 in both the CB2 and the chimera, and M265 in the CB2 and its corresponding M278 in the chimera are highlighted in yellow; I186 and W194 in both the CB2 and the chimera are highlighted in blue; V261 in the CB2 is highlighted in gray.

On the other hand, we found that the chimeric CB1/2 receptor was activated by WIN55,212-2 in a concentration-dependent manner and showed a CB2-like functional property. This similarity can be explained based on the cryo-EM structure analysis of CB2-Gi bound to WIN55,212-2 published by Changrui Xing et al. [38]. As the structural analysis reveals (Figure 4), the naphthalene moiety of WIN55,212-2 extends between TMHII and TMHIII and is predicted to form strong π-π interactions with F91 and F94 and hydrophobic interactions with F87, H95, P184, and F281. Also, the core structure of WIN55,212-2 (2,3-dihydro-[1,4]oxazino[2,3,4-hi]indole) points downward and engages in π-π interactions with F117 and W258. It also interacts with I110, V113, F183, V261, and M265 via hydrophobic interactions, which have been shown previously to play key roles in the ligand binding of the CB2 receptor. Furthermore, the morpholine moiety of WIN55,212-2, which adopts the chair conformation, approaches TMHV and ECL2 to form additional hydrophobic interactions with critical residues that have been reported to function in ligand binding, including F183, I186, and W194 [38].

Among the above mentioned amino acid residues, W258 is a highly conserved residue in class A GPCRs (Class A, also known as the “rhodopsin-like family”, is the largest group of GPCRs and accounts for around 80% of GPCRs [39]), and has been reported to have a crucial role in GPCR activation [40], and the three residues, W194, F117, and W258, were found to potentially play essential roles in distinguishing the agonist from the antagonist in CB2 receptors [38]. These above mentioned critical amino acid residues of CB2 receptors responsible for the binding of WIN55,212-2 are all conserved in the chimeric CB1/2 receptor, except for V261, which corresponds to L274 in the chimera, indicating that the chimeric CB1/2 receptor highly preserves the CB2 functional property for the activity of WIN55,212-2. This CB2-like property is possibly the reason of the comparable I_K,WIN_ responses and the similar dual modulatory effect observed in chimeric CB1/2- and CB2-GIRK1/GIRK2-RGS4 coupling systems.

The abolishment of OS activity and the CB2-like functional characteristics of the chimeric CB1/2 receptor for WIN55,212-2 activity suggest that the important amino acid residues for the binding of OS to CB2 receptors are different from those for the binding of WIN55,212-2, which is in line with the OS-induced non-competitive antagonism on CB2 receptors in the presence of WIN55,212-2. Since the chimeric CB1/2 receptor shows a CB2-like functional property, the TMHV-ICL3-TMHVI domain of the CB1 receptor does not seem to play an essential role in OS activity in the chimeric CB1/2 receptor.

The in vivo evaluation of the therapeutic potential of OS was conducted in mice with a battery of behavior tests, including cage activity [30], open field (OF) [30], elevated plus maze (EPM) [31], and Morris’ water maze (MWM) [32]. CB1 receptor activation has been consistently discovered to induce learning and memory deficits in rodents, with the overall result that both acute and repeated administration of Δ9-tetrahydrocannabinol (THC), HU210, WIN55,212-2 CP-55,940, or anandamide (AEA) impaired learning and memory [41,42,43,44,45]. Moreover, several CB1 antagonists can improve cognitive deficits and ameliorate spatial learning and memory impairment, such as AM-251 and AM-281 [3].

In this study, we observed a significant learning and memory impairment in mice following WIN55,212-2 injection in the MWM test (Figure 3H), which is consistent with previously published results. Moreover, OS counteracted learning and memory deficit caused by WIN in mice, which is consistent with our result that OS blocked CB1-dependent K^+^ currents evoked by WIN in the ex vivo CB1-GIRK1/GIRK2-RGS4 coupling system and with the above mentioned effects of other CB1 antagonists.

Also, we did not see significant side effects of OS on locomotion or anxiety-like behavior in our animal experiments. This is important given that both tests (OF and EPM) show that animals did not present any locomotion or anxiety-like state alteration during the learning and memory tests that would follow. Combined with our selectivity screening via electrophysiological assays, OS, being a natural molecule, seems to be unlikely to possess severe side effects, at least in locomotion or anxiety state.

Although our study is a precursor in the use of OS as a preventive treatment for learning and memory deficits caused by CB agonists, future studies, such as CB1/2 assessment in regions involved in memory and behavior, such as in the hippocampus, are required to elucidate the real effect, mechanisms, and pathways through which OS acts in vivo. Nonetheless, our electrophysiological results express what happens at the receptor level and are consistent with our in vivo findings and previous research.

The exact mechanism behind the anxiolytic effects of OS, whether through direct actions on CB receptors or other off-target mechanism(s), requires further exploration. Comprehensive selectivity screening and in-depth in vivo studies will be necessary to fully understand the targets of OS, which will be valuable for assessing its therapeutic potential and possible side effects. Future research efforts can also focus on optimizing OS to enhance its potency and selectivity. Like the OS compound, rimonabant is also a CB1 receptor antagonist, indicated for the treatment of obesity and related metabolic risk factors in nondiabetic and diabetic overweight and obese patients [46,47]. However, this compound was withdrawn from the market due to psychiatric adverse effects such as depression and even suicidal ideation [11,12]. These side effects are linked to the central psychiatric adverse effects of the CB1 receptor signaling pathway [11,12]. At present, we cannot exclude that OS too would suffer from similar side effects as rimonabant, limiting its potential to become a drug.

One point to be considered is that animal venoms constitute a natural library of several million molecules, largely unexplored at present as a source of potential drugs. Right now, only six venom-derived drugs are available on the market [43], with many other candidates in clinical development and hundreds of patents being filed, highlighting their therapeutic potential [43]. In this sense, our results show that OS could be potentially added to this list as a promising candidate for learning and memory deficits in neurological diseases that involve, at least, the cannabinoid system.

## Data Availability

Data, material, and software information are provided as supporting information (SI) and in the article.

## Supplementary Materials

The following supporting information can be downloaded at: https://www.mdpi.com/article/10.3390/biomedicines12020454/s1, Figure S1: The structures of oleoyl serotonin (OS) found in the venom of Stephanoconus snails, endocannabinoid AEA, and the allosteric modulator of the CB1 receptor—Org27569; Figure S2: The best fit for the concentration–activation curve of effects of WIN55,212-2 on the CB-GIRK1/GIRK2-RGS4 coupling system; Figure S3: Representative electrophysiological current traces show the effect of OS on ion currents in oocytes expressing different receptors and channels.
