# Supplementary material for: The Sobering Sting: Oleoyl Serotonin Is a Novel Stephanoconus Snail Venom-Derived Antagonist of Cannabinoid Receptors That Counteracts Learning and Memory Deficits
Source: Biomedicines. 2024 Feb 18;12(2):454. doi: 10.3390/biomedicines12020454 (PMC10887214; doi:10.3390/biomedicines12020454)
Supplement: Supplementary file 1 [file biomedicines-12-00454-s001.zip › biomedicines-2811757-supplementary.pdf]

Supporting Information for

The sobering sting: A novel cone snail venom-derived antagonist of cannabinoid receptors counteracts learning and memory deficits.

Dongchen An[a,1], Guilherme Salgado Carrazoni[b,1], Ben-Hur Souto das Neves[b], Rudi D'Hooge[b,*], Steve Peigneur[a,*], Jan Tytgat[a,*].

[a]Toxicology and Pharmacology, KU Leuven Campus Gasthuisberg, ON2, Herestraat 49, Box-922, 3000 Leuven, Belgium.

[b]Laboratory of Biological Psychology, KU Leuven, Tiensestraat 102, Box-3714, 3000 Leuven, Belgium.

[1]Dongchen An and Guilherme Salgado Carrazoni contributed equally to this work.

*Corresponding authors: Rudi D'Hooge, Steve Peigneur, Jan Tytgat.
Emails: rudi.dhooge@kuleuven.be. steve.peigneur@kuleuven.be, jan.tytgat@kuleuven.be.

This PDF file includes:

Figures S1 to S3

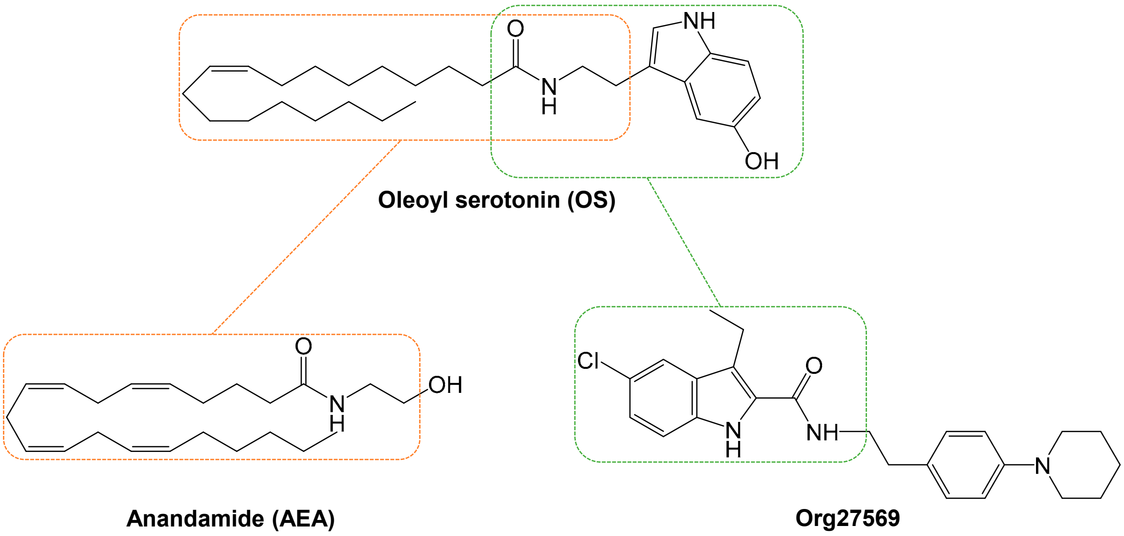

Fig. S1. The structures of oleoyl serotonin (OS) found in the venom of *Stephanoconus* snails, endocannabinoid AEA, and the allosteric modulator of the CB1 receptor—Org27569.

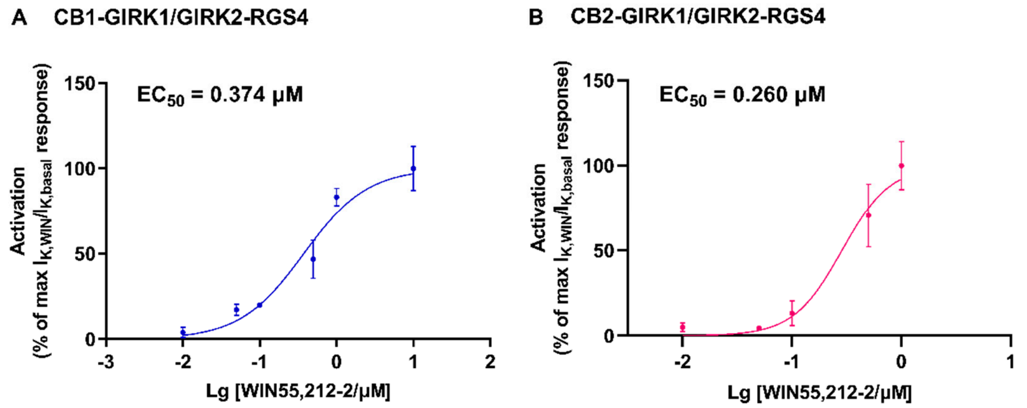

Fig. S2. The best fit for the concentration-activation curve of effects of WIN55,212-2 on the CB-GIRK1/GIRK2-RGS4 coupling system. (A) CB1 receptor activation, the blue curve, and (B) CB2 receptor activation, the magenta curve, were evoked on the application of a range of different concentrations of WIN55,212-2 in the presence of HK. Each data point represents the mean ± SD of three determinations from two or three batches of oocytes (n = 3). This figure is cited from authors' previous publication (5).

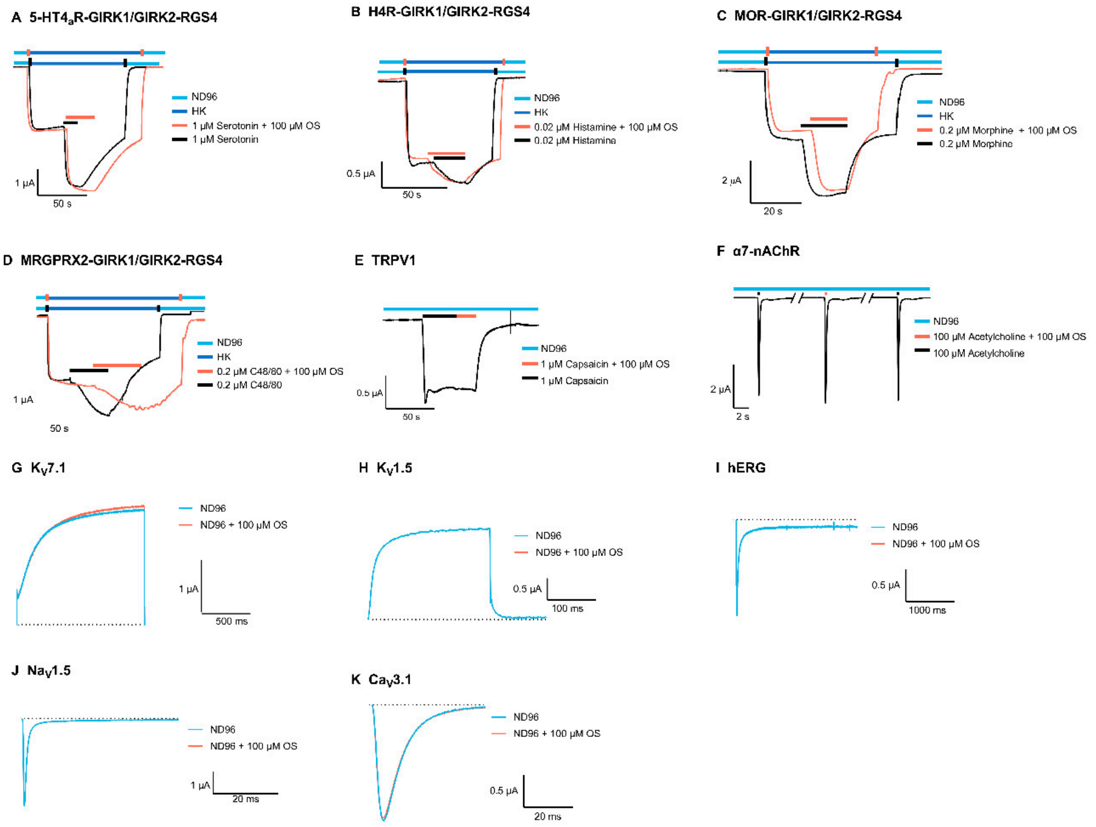

Fig. S3. Representative electrophysiological current traces show the effect of OS on ion currents in oocytes expressing different receptors and channels (N = 3). (A), (B), (C), and (D) represent that the GPCR agonist + 100 μM OS (orange trace) and the agonist alone (black trace) were applied to GPCR-GIRK1/GIRK2-RGS4 coupling systems expressed in oocytes following $I_{K,basal}$. OS did not significantly change the response of receptor-dependent inward K^+ currents induced by the GPCR agonists. (E) and (F) represent that the agonist + 100 μM OS was applied to TRPV1 and α7-nAChR expressed in oocytes following the agonist-evoked inward currents through the channel and the receptor, and OS did not significantly change the response of inward currents induced by the agonists. (G), (H), and (I) represent that the 100 μM OS was applied to the K_V7.1, K_V1.5, and hERG channel expressed in oocytes, and OS did not significantly change the response of potassium currents through the channels. (J) represent that the 100 μM OS was applied to the Na_V1.5 channel expressed in oocytes, and OS did not significantly change the response of the sodium current. (K) represents that the 100 μM OS was applied to the Ca_V3.1 channel, and OS did not significantly change the response of the calcium current. The concentrations of all the agonists were approximately their corresponding EC_{50} values for activating the receptors and channels expressed in oocytes, as described before (6-8). All panels are representative of at least three independent experiments (n ≥ 3).
